# Supplementary material for: Comprehensive Knowledge and Preparedness among Dental Community to Confront COVID-19—A Multicentric Cross-Sectional Study
Source: Int J Environ Res Public Health. 2021 Dec 25;19(1):210. doi: 10.3390/ijerph19010210 (PMC8751054; doi:10.3390/ijerph19010210)
Supplement: Supplementary file 1 [file ijerph-19-00210-s001.zip › ijerph-1501126-supplementary.pdf]

Questionnaire

Sl No.	Demographic Details	
**1.**	**Age Group**	a. Less than 20<br>b. 20-29 years<br>c. 30–39 years<br>d. 40–49 years<br>e. >50 years
**2.**	**Gender**	a. Male<br>b. Female
**3.**	**Duration of practice**	a. <5 years<br>b. 5–15 years<br>c. >15 years
**4.**	**City of practice**	
**5.**	**Occupation**	**General Dentist**<br>**Dental Specialist**<br>a. Oral medicine<br>b. Pedodontics<br>c. Periodontics<br>d. Prosthodontics<br>e. Restorative dentistry<br>f. Oral Surgery<br>g. Oral Pathology<br><br>**Dental Student**<br>a. Pre-clinical course<br>b. Clinical course<br>c. Interns<br>d. Post-graduate and above
**6.**	**Organization**	a. Private sector<br>b. Public sector<br>c. University Clinics
**7.**	**Educational status**	a. BDS<br>b. MDS<br>c. Doctoral (PhD)
**8.**	**Training taken (Infection control)**	a. Yes<br>b. No
**9.**	**Training taken (COVID-19)**	a. Yes<br>b. No

**10.**	**Nationality**	

Sl No.	**Knowledge Questions**	
**1.**	**Which of the following is the best way to prevent COVID-19?**	a. Washing hands<br>b. Wearing mask<br>c. Using gloves<br>d. Using disinfectant
**2.**	**In which the following case is the virus alive more?**	a. Steel and metal surfaces<br>b. Body skin<br>c. Clothing surface<br>d. Water
**3.**	**Which ones are the main symptoms of COVID-19?**	a. The feeling of pain or constant pressure in the chest<br>b. Difficulty in breathing or shortness of breath<br>c. Bruising of the lips or face<br>d. Confusion and irritability as a new event<br>e. Loss of sense of taste or smell<br>f. Gastrointestinal symptoms such as diarrhea and vomiting
**4.**	**What is the most definitive diagnostic procedure of COVID-19?**	a. Chest CT<br>b. Ultrasound<br>c. PCR<br>d. Urine sample
**5.**	**When should the N95 mask's valve be closed?**	a. During inhaling<br>b. During exhaling<br>c. both states
**6.**	**What is the best and most effective way to disinfect surfaces in dental offices and clinics?**	a. Disinfectants with alcohol 70%<br>b. Quaternary ammonium<br>c. Sodium hypochlorite with a concentration below 0.5%
7.	**In case of a lack of facilities, which of the following cases is necessary to reduce the transmission of coronavirus?**	a. Long-sleeve and waterproof cover<br>b. Surgical cap and gloves<br>c. FFP2 or N95 standard mask or their equivalent<br>d. Eye care equipment (shield or safety glasses)
8.	**Which group of patients do you think should avoid coming to the dental offices during the**	a. Children<br>b. Elderly<br>c. Breastfeeding and pregnant women

	**corona pandemic, except during emergencies?**	d. People with systemic problems
9.	**Rate of spread of which disease is the highest?**	a. COVID19<br>b. H1N1<br>c. Measles<br>d. HIV
10.	**Which one has the most effective role in reducing the prevalence of corona?**	a. Social distancing<br>b. Cleansing the surface<br>c. Airway protection<br>d. Disinfecting the nasal passage by normal saline
11.	**What is the main route of the contagion of the SARS-COV2 virus?**	a. Blood<br>b. Breathing<br>c. Oral-fecal<br>d. Skin contact
12.	**What is the appropriate distance between people to prevent the contagion of COVID-19?**	a. At least 1 meter<br>b. At least 3 meters<br>c. At least 2 meters<br>d. People should not have contact
13.	**What is the best kind of gloves for resistance against coronavirus?**	a. Latex<br>b. Vinyl<br>c. Nitrile<br>d. Nylon
14.	**In general, for how long are the N95 and FFP2 filter masks useful?**	a. 1 hour<br>b. 8 hours<br>c. 24 hours<br>d. One week
15.	**Is it necessary to use any pre procedural mouthwash while treating patients during COVID- 19?**	a. Yes<br>b. No<br>c. I don't know<br>d. Please specify the name of the mouthwash................
16.	**Is it necessary to use a protective shield while treating patients?**	a. Yes<br>b. No<br>c. I don't know
17.	**Is it necessary to wash our hands before wearing gloves?**	a. Yes<br>b. No<br>c. I don't know
18.	**How long do you take to wash your hands?**	a. I don't wash hands before wearing gloves<br>b. Less than 2 minutes<br>c. 2-5 minutes

		d. 5 minutes or more
19.	**How often do you change mouth masks?**	a. After every patient<br>b. When mask is damaged<br>c. After every session<br>d. When mask is dampened

Sl No.	**Preparedness Questions**	
1.	**Personal protection measures**	a. Social distancing (avoid personal contact)<br>b. Hand hygiene (washing hands more often)<br>c. Use face mask (cover nose)<br>d. Avoid travel to infected area or country<br>e. Avoid visiting wet markets, raw and under cooked animal products<br>f. Use different chopping board and knives for raw meat and other food<br>g. Avoid purchasing things made in china<br>h. Avoid vegetarian food<br>i. Don't know
2.	**Treatment available for COVID-19**	a. No treatment/vaccine till date<br>b. Supportive treatment<br>c. Just keep yourself safe<br>d. Vaccination<br>e. Don't know
3.	**Preparedness to fight against COVID-19**	a. Avoiding mass gathering and traveling to suspected area<br>b. Using hand sanitizer, face mask, home cleaning materials recent days<br>c. Spending 20 s thoroughly for washing hands now a days<br>d. Maintaining food hygiene<br>e. Stored food items and basic required things in home as its lockdown<br>f. Ready to visit hospital immediately if needed
4.	**Do you need more information about prevention of COVID 19?**	a. Yes<br>b. No
5.	**In terms of infection control, how did/should you prepare yourself to deal with COVID-19 infection**	a. Check adequate supplies of goggles, masks, and gowns on hand for emergencies.

		b. Links to orcontact External Resource Centers for COVID-19 (Coronavirus) (CDC, WHO etc.).<br>c. Check patient care equipment, including portable ventilators.<br>d. Recommendations for infection control to help biomedical and clinical engineers.<br>e. Check alternative suppliers of certain personal protective equipment.<br>f. Prepared the list to supply chain professionals.<br>g. Do not need any preparation.<br>h. I don't know.
6.	**Dentist should provide adequate training to their staff to promote many levels of screening and preventive measures.**	a. Agree<br>b. Disagree<br>c. Do not know
7.	**What precautions should the dental practitioners take when treating COVID-19-positive patients?**	a. Airborne infection isolation rooms (AIIRs) should be reserved for patients<br>b. Air from these rooms should be extracted directly by high-efficiency particulate air (HEPA) filter<br>c. Pre-procedural mouth rinse with 0.2% povidone iodine<br>d. Extra-oral imaging should be preferred to intra-oral imaging<br>e. Rubber dam should be used to minimize splatter generation<br>f. Minimize the use of ultrasonic instruments, high-speed headpieces, and 3-way syringes.
8.	**Do you know whom to contact if you have an unprotected exposure to a known or suspected COVID-19 patient?**	a. Yes<br>b. No
9.	**Do you know what to do if you have signs or symptoms suspected of COVID-19 infection?**	a. Yes<br>b. No
10.	**During COVID-19 pandemic, how do you recap the needle after use?**	a. One handed recapping<br>b. Two handed recapping<br>c. I do not recap a used needle

11.	**How many needle-stick injuries have you had since COVID-19 pandemic?**	a. 1–5<br>b. 6–10<br>c. > 10<br>d. Not applicable
12.	**During COVID-19 pandemic, is it advised to encourage the wound to bleed as the initial action following sharp injury?**	a. Yes<br>b. No<br>c. Don't know
13.	**During COVID-19 pandemic, what actions do you most commonly take following a needle stick or sharp injury in the clinic?**	a. Ignore and continue Replace gloves<br>b. replace needle<br>c. Inform nurse/supervisor<br>d. Follow each step of college policy
14.	**Are you aware of your college sharps policy and procedures?**	a. Fully aware of policy and procedures<br>b. Aware policy exists but unaware of procedures<br>c. Unaware of policy or procedures
